# Isolation of Cellulose Nanocrystals from Banana Peel Using One-Pot Microwave and Mild Oxidative Hydrolysis System

**DOI:** 10.3390/nano12193537

**Published:** 2022-10-10

**Authors:** Nurhidayah Azmirah Mohd Jamil, Syafiqah Syazwani Jaffar, Suryani Saallah, Mailin Misson, Shafiquzzaman Siddiquee, Jumardi Roslan, Wuled Lenggoro

**Affiliations:** 1 Biotechnology Research Institute, Universiti Malaysia Sabah, Jalan UMS, Kota Kinabalu 88400, Sabah, Malaysia; 2 Marine Aquaculture Development Centre Menggatal, Department of Fisheries Sabah, Jalan Sepanggar, Kota Kinabalu 88450, Sabah, Malaysia; 3 Faculty of Food Science and Nutrition, Universiti Malaysia Sabah, Jalan UMS, Kota Kinabalu 88400, Sabah, Malaysia; 4 Institute of Engineering, Tokyo University of Agriculture and Technology, 2-24-16 Nakacho, Koganei, Tokyo 184-8588, Japan

**Keywords:** nanocellulose, banana peel, one-pot, microwave, hydrogen peroxide, acid hydrolysis

## Abstract

The current investigation deals with the application of a one-pot system to facilitate the production of cellulose nanocrystals (CNCs) from banana peel by a combination of microwave pre-treatment and mild oxidative hydrolysis with hydrogen peroxide (H_2_O_2_, 0–30 wt%) and sulfuric acid (H_2_SO_4_, 0–10%). H_2_O_2_ causes decolorization of the banana peel suspension from dark brown to light yellow, while further treatment with H_2_SO_4_ produces a white suspension, indicating successful removal of the non-cellulosic components from the banana peel. This finding was further supported by Fourier Transform Infrared (FTIR) spectroscopic analysis, which showed the gradual disappearance of lignin and hemicellulose peaks with increasing H_2_O_2_ and H_2_SO_4_ concentrations. The CNCs has considerably high crystallinity, with the highest crystallinity (~85%) being obtained at 6% H_2_SO_4_. Therefore, CNCs obtained at 6% H_2_SO_4_ were selected for further characterization. Scanning Electron Microscope (SEM) analysis confirmed the disintegration of the cellulose fibres into small fragments after hydrolysis. Transmission Electron Microscope (TEM) and Atomic Force Microscope (AFM) analyses revealed the spherical shape of the CNCs with an average size of approximately 20 nm. The CNCs have good stability with zeta potential of −42.9 mV. Findings from this study suggest that the combination of microwave pre-treatment and oxidative hydrolysis with 30 wt% H_2_O_2_ and 6% H_2_SO_4_, which is about 11 times lower than the commonly used H_2_SO_4_ concentration, is proven effective for the isolation of CNCs from banana peel. These observations are expected to provide insight into a facile and environmentally benign alternative to the conventional CNCs isolation method, using abundant and underutilized agricultural waste as feedstock.

## 1. Introduction

Many efforts have been made in the development of eco-friendly materials for a variety of purposes throughout the past century. Among these materials, cellulose stands out due to its abundance, light weight, recyclability, and biodegradability [1]. However, despite their abundance, woods and croplands have a finite capacity to provide cellulosic resources, a reality that urges researchers and industries to find alternative sources in order to maximize the efficiency of natural resource exploitation. In this regard, agricultural wastes, by-products of agricultural activities such as rice husk [2], rice straw [3], corn cob [4], coconut shell [5], orange tree pruning [6], and fruit peels such as pear [7] and banana [8], are seen as promising lignocellulosic feedstock for various applications.

Bananas (*Musa sp.*) are a staple food crop that are widely grown in Asia, Latin America, and Africa, playing a significant role in food security and economy. The global production of bananas is more than 100 million metric tons (MMT) per year and accounts for 16% of the total global food production [9]. The high consumption and industrial processing of the edible parts of the banana, however, results in a vast amount of waste, including peel, which is often dumped in landfills and creates negative environmental impact [8,10,11] due to microbial decomposition and emission of greenhouse gases such as methane and carbon dioxide [12,13]. Given that banana peel contains a considerable amount of cellulose (7.5–18.7%) [14,15,16,17,18,19,20], conversion of this lignocellulosic biomass to nanocellulose in the form of cellulose nanocrystals (CNCs) or cellulose nanofibers (CNFs) could add value to the underutilized by-products as well as reduce environmental pollution, in line with the circular economy concept and Sustainable Development Goals (SDG) [12].

Nanocellulose is a sustainable nanomaterial that has become a topic of great interest in recent years due to the inherent cellulose properties (i.e., most abundant in nature, renewable, biodegradable, low toxicity) and outstanding features such as high specific surface area, high aspect ratio and superior mechanical strength [21,22,23,24,25]. Its good barrier properties and tunable surface chemistry are particularly attractive in food science and technology applications such as food packaging, additives, and stabilizers [26,27]. Pelissari et al. [16] and Tibolla et al. [15] have reported that the extraction of nanocellulose from banana peel was possible through several pre-treatments steps, including repeated alkalization (5% KOH, 14 h) and bleaching (1% NaClO_2_, 1 h, 70 °C), followed by successive washing to prepare relatively pure cellulosic starting materials prior to hydrolysis with dilute sulfuric acid (H_2_SO_4_, 0.1–10%, 80 °C, 1 h) and high-pressure homogenization (0–7 passages). However, such multistep extraction procedures are laborious, time-consuming, and involved the use of harmful chemicals, high water consumption for neutralization, and high energy, which become an obstacle for the industrialization and application of nanocellulose [28]. Therefore, innovative solutions in nanocellulose isolation method are urgently needed to overcome these drawbacks.

Harini et al. [8] implemented the combination of microwave and hydrogen peroxide (H_2_O_2_) as a green alternative to the conventional chemical pre-treatment to afford the production of cellulose microfibers from banana peel. Microwave provides high heating efficiency, lower energy requirements, faster treatment time, selective processing, and easy operation. H_2_O_2_ is a powerful oxidizing agent that decomposes into water and oxygen, thus creating no harmful by-product [29]. A combination of microwave and H_2_O_2_ could therefore promote effective and rapid removal of the non-cellulosic components without the need for alkaline treatment [8]. Interestingly, the use of H_2_O_2_ eliminate the needs for successive washing and enables the acid hydrolysis reaction to be conducted in one-pot, as recently reported by Chen et al. [7] and Chávez-Guerrero et al. [30]. However, the combination of microwave and oxidative hydrolysis with H_2_O_2_ and dilute H_2_SO_4_ in one-pot for nanocellulose isolation has never been reported, hence the objective of the present study. The effect of H_2_O_2_ and H_2_SO_4_ concentrations in the range of 0–30% and 0–10%, respectively, on the nanocellulose production was investigated. The nanocellulose produced were characterized for their chemical functionality, crystallinity, morphology, size, and colloidal stability by FTIR, XRD, SEM, TEM, AFM, and zeta potential analyses.

## 2. Materials and Methods

### 2.1. Materials

Bananas (*Musa acuminata* × *balbisiana*) with maturity stage 7 (yellow peel with little brown spot) were purchased from a local market in Kota Kinabalu, Sabah, Malaysia, and immediately processed in the laboratory. Hydrogen peroxide (H_2_O_2_, 30%) and sulfuric acid (H_2_SO_4_, 98%) were a product of Merck, Germany. A cellulose standard, Avicel^®^ PH-101 (microcrystalline), and sodium metabisulfite (Na_2_S_2_O_5_, 97%) were supplied by Sigma-Aldrich. Deionized water (Millipore) was used throughout this work. All of the reagents used were of analytical grade.

### 2.2. Preparation of Banana Peel (BP) Powder

The banana peel (BP) was removed from its flesh and immersed in 2% sodium metabisulphite solution for 24 h to avoid oxidation and microbial spoilage. The peel was then dried in an electric cabinet dryer (Shin-1) for 48 h at 60 °C. The dried peel was cooled to room temperature before being grinded by a mixer grinder (MX-AC2105, Panasonic Malaysia Sdn. Bhd., Selangor, Malaysia) and sieved with 250 πm laboratory test sieve (Endecotts, BS-410-1, London, UK) to obtain fine powder. The BP powder was then kept in an airtight container at room temperature until further use.

### 2.3. Proximate and Chemical Composition Analyses

The BP powder was analyzed for its proximate composition according to the Association of Official Analytical Chemists (AOAC, 2005) procedure [31]. Determination of moisture content was carried out using an oven dry method at 105 °C for 12 h until a constant weight was reached, while the ash content was measured using a muffle furnace ashing method in which the samples were heated in a furnace at 550 °C for 8–12 h. Crude protein content was determined using protein analyzer (Kjeltec^®^2300 Analyzer Unit, Hillerod, Denmark) following the Kjeldahl method. The fat content was analyzed using a solvent extraction system (Soxtec^®^Avanti 2050 Auto System, Höganäs, Sweden). Lignocellulosic fractions of the BP powder, including cellulose, hemicellulose, and lignin, were analyzed according to the Technical Association of the Pulp and Paper Industry (TAPPI) standard methods, as described by Song et al. [32]. All the analyses were performed in triplicates and the results were reported as average.

### 2.4. Isolation of CNCs from BP Powder by One-Pot Process

**Figure 1:**
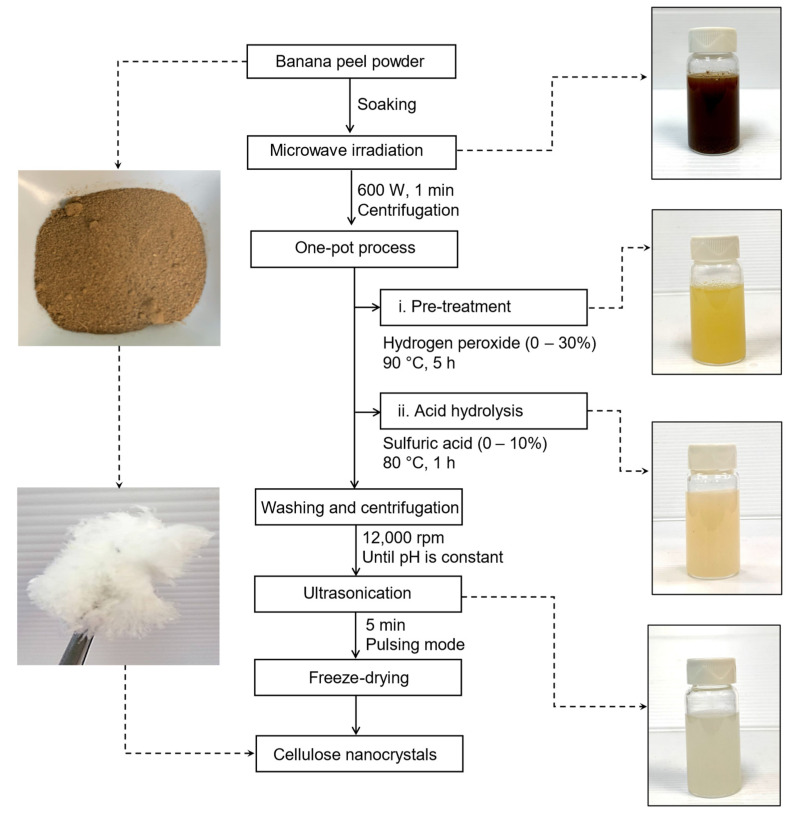
Step-by-step procedures for isolation of CNCs from banana peel.

Production of CNCs from the BP powder was conducted by using a modified one-pot method [33]. Briefly, 10 g of the dried BP powder was soaked in deionized water and subjected to microwave irradiation at 600 W for 1 min to loosen the compact lignocellulosic structure. The swollen BP was then centrifuged and filtered to remove water from the sample. Bleaching was performed by the addition of 100 mL H_2_O_2_ to the same pot with a concentration ranging from 0 to 30% followed by 5 h incubation at 90 °C with mechanical agitation. After that, the reaction mixture was cooled to room temperature in an ice bath. Hydrolysis was performed by adding dilute H_2_SO_4_ with a concentration ranging from 0 to 10% into the pot containing the suspension and heated to 80 °C for 1 h. The mixture was quenched with ice cubes to stop the reaction. In order to remove the non-fibrillated cellulose components, successive washing with deionized water and centrifugation at 12,000 rpm (25 °C) were conducted until a constant pH was reached. The suspension was then ultrasonicated in a pulsing mode (15 s on and 5 s off) for 5 min to reduce agglomeration and improve nanocellulose dispersibility. The resultant suspension was freeze-dried and kept in a sealed container at 4 °C until further use. The procedures for the modified one-pot nanocellulose isolation are depicted in Figure 1. The yield of CNCs was determined using Equation (1):

Yield (%) = (W_CNCs_/W_BP_) × 100 (1)

where W_CNCs_ is the dry weight of CNCs obtained after freeze-drying and W_BP_ is the dry weight of the banana peel powder [15].

### 2.5. Fourier Transform Infrared Spectroscopy (FTIR)

Changes in chemical compositions of the BP powder after hydrogen peroxide pre-treatment and dilute sulfuric acid hydrolysis were monitored using Fourier-Transform Infrared Spectroscopy (FTIR). Agilent Cary 630 FTIR Spectrometer (Agilent Technologies, Inc., Danbury, CT, USA) was used to collect the absorbance spectra in the infrared region between 4000 and 600 cm^−1^ with a spectral resolution of 4 cm^−1^ and 32 scans. The measurement was conducted at room temperature. Avicel^®^ PH-101 microcrystalline cellulose (Sigma Aldrich) was used as a reference [34].

### 2.6. X-ray Diffraction (XRD) Analysis

XRD pattern of the dried samples were recorded using a Rigaku SmartLab X-ray diffractometer (Rigaku Corporation, Tokyo, Japan). The analysis was performed at 25 °C, with an X-ray generator of 40 kV and 50 mA in the diffraction angle (2θ) range 5° to 80°. Crystallinity index (I_CR_) was determined using Equation (2) [7,15]:

I_CR_ (%) = [(I_200_ − I_am_)/ I_200_] × 100 (2)

where I_200_ is the maximum intensity of the diffraction peak from the (200) plane at 2θ ≈ 22° and I_am_ is the intensity of amorphous region between the (110) and (200) planes (2θ ≈ 18°). The CNCs sample with highest crystallinity was selected for further characterizations.

### 2.7. Scanning Electron Microscope (SEM) Analysis

Morphological properties of the BP powder and the resulting cellulose and CNCs were examined using a Scanning Electron Microscope (SEM). The samples were placed on a stub by using double-sided black conducting tape and observed by SEM under vacuum at an accelerating voltage of 10 kV. The samples were sputter-coated with gold prior to the analysis.

### 2.8. Atomic Force Microscope (AFM) Analysis

Topographic imaging of the samples was performed using Dimension Icon AFM instrument (Bruker, Santa Barbara, CA, USA). The scanning speed and area were 0.6 line/s and 0.5 × 0.5 μm^2^, respectively. Before being subjected to AFM imaging, dilute suspension (0.01 wt%) were first prepared followed by 5 min ultrasonication. One drop of the sonicated suspension was dispersed on a freshly cleaned optical glass substrate and air-dried at room temperature. 

### 2.9. Transmission Electron Microscopy (TEM)

The image was acquired by using a Tecnai G2 Spirit BioTWIN transmission electron microscope (TEM) (FEI, Hillsboro, OR, USA) with an operating voltage of 80 kV. Prior to the analysis, the dilute CNCs suspension was ultrasonicated for 5 min. Then, a drop of suspension was placed on a carbon microgrid (300 mesh) and dried at 60 °C for 20 min.

### 2.10. Particle Size and Zeta Potential Measurements

Particle size and zeta potential of the CNCs suspension (0.1 wt% in deionized water) were determined using NanoPlus Particulate Systems (Micromeritics, Norcross, GA, USA) under the following conditions: water refractive index 1.3328, viscosity 0.8878 cP, dielectric constant 78.30, temperature 25 °C. Before analysis, the suspension was ultrasonicated for 5 min to improve sample dispersibility. The result is presented as the average value of three measurements.

## 3. Results and Discussion

### 3.1. Proximate and Chemical Compositions of the BP Powder

Table 1 shows the proximate compositions of the BP powder, which include moisture, ash, fat, and protein contents. The BP powder has a moisture content of 8.2 ± 0.2%, comparable to those of the *Musa sapientum* Linn. cv. Mali-Ong (7.7%) banana peel [29]. Higher moisture content (15.5–17.8%) was reported by Ibiyinka et al. [35] for ripe *Musa sapientum* banana peels from different varieties (cavendish, nino, and red banana). Moisture content plays a critical role in determining the storage stability of the product. A product with low moisture content can be stored longer, as it is less prone to microbial degradation and chemical changes [36]. Considerably high ash content (13.8 ± 0.3%) was obtained, corroborated with the findings of Singanusong et al. [29] and Deb et al. [37], which indicate a high mineral content in the BP powder. The high fat content (12.0 ± 0.2%) could be associated with the synthesis of fatty acids during fruit metabolism., in accordance with the data reported by Deb et al. [37] for *Musa acuminata* banana peel. The protein content (5.4 ± 0.1%) obtained here was almost similar to that of the peel of *Musa sapientum*, as reported by Pyar and Peh [36].

The chemical compositions of the BP powder are shown in Table 2 and compared to those obtained by other researchers. The cellulose, hemicellulose, and lignin contents in the BP powder were found to be 12.1 ± 0.3%, 14.8 ± 0.9%, and 15.7 ± 2.1%, respectively, indicating that the BP powder has a considerable amount of cellulose that can be utilized for CNCs production. The cellulose and hemicellulose content obtained is in accordance with those reported by Tibolla et al. [15] for Terra banana peel. In an earlier study by Tibolla et al. [14], lower cellulose content was observed for the same banana variety. The BP powder has comparatively lower hemicellulose content and higher lignin content than other banana peels, as displayed in Table 2. These findings imply that the both the proximate and chemical compositions in banana peel are not just influenced by the banana variety, but also other factors, such as state of ripeness, soil, and climate condition during harvesting [38,39].

### 3.2. Isolation of CNCs from BP Powder by One-Pot Process

The BP powder was successfully converted into CNCs with a yield of 28.1% by using a one-pot process of microwave pre-treatment and oxidative hydrolysis. Throughout these processes, the non-cellulosic components from the BP powder were removed, leaving a white “sugar candy” appearance of CNCs after freeze-drying (Figure 1). The yield of CNCs obtained is comparable to the yield reported by Tibolla et al. [15] (27.1%) for CNFs produced from banana peel using the conventional chemical pre-treatment with KOH and NaOCl_2_, followed by hydrolysis with 10% H_2_SO_4_ and five passages of high-pressure homogenization. Other studies have reported a lower yield of CNCs produced using the typical procedures of alkalization, bleaching, and H_2_SO_4_ hydrolysis, as stated by Chen et al. [7].

**Figure 2:**
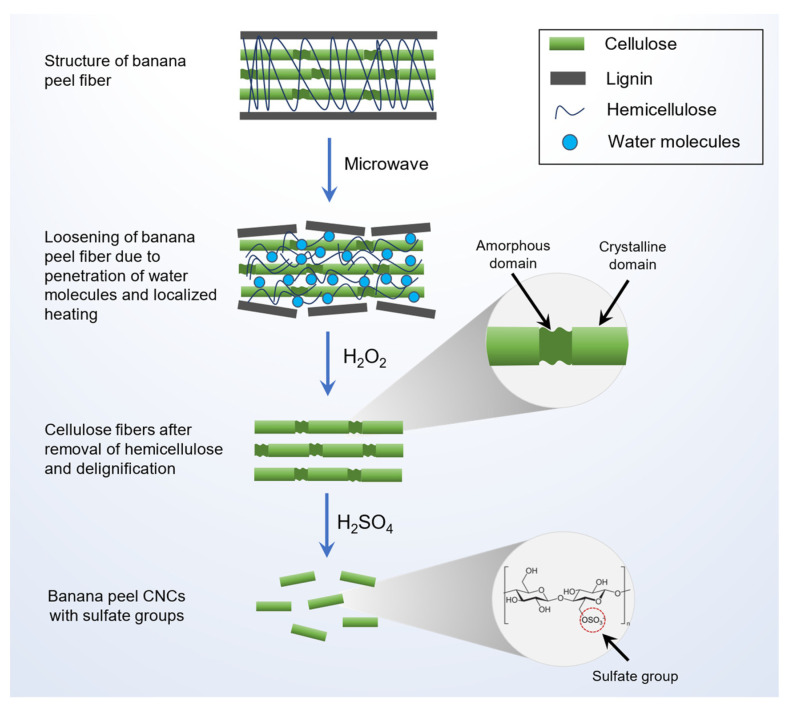
Mechanism of CNCs formation through one-pot microwave and oxidative hydrolysis.

The mechanism of the one-pot process employed in the present study is depicted in Figure 2. The implementation of one-pot process does not just simplify the practical aspects of the CNCs isolation, but also saves time and minimizes product losses between steps [7,30]. By applying the microwave pre-treatment, the time required for overnight soaking and conventional heating could be reduced. Dielectric polarization by microwave energy facilitates rapid loosening and swelling of the lignocellulosic structure by penetration of water molecules in the space between the cellulose microfibers [8,40]. Further pre-treatment using H_2_O_2_ facilitates the removal of hemicellulose and the delignification process, enabling the implementation of the one-pot process as the decomposition of hydrogen peroxide produced water and oxygen [41,42]. Thus, successive washing after pre-treatment is unnecessary, and hydrolysis can be carried out directly in the same pot. In this way, water consumption for neutralization can be minimized effectively.

#### 3.2.1. Effect of Hydrogen Peroxide Concentration on the Pre-Treatment of BP Powder

**Figure 3:**
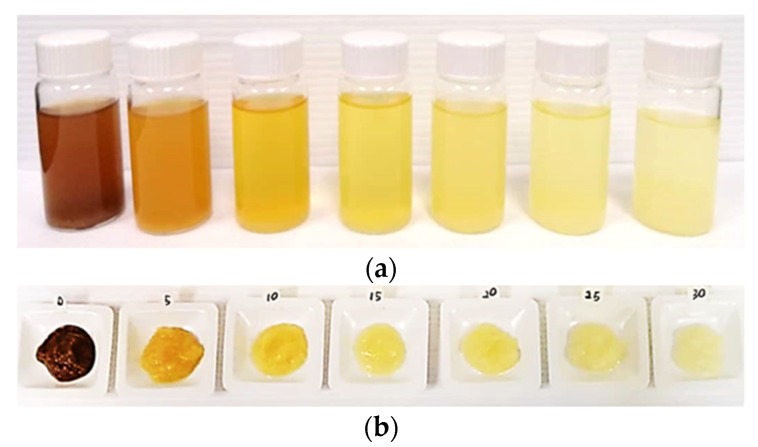
Washing supernatants (**a**) and insoluble residues (**b**) obtained after pre-treatment with hydrogen peroxide at concentration ranging from 0% (control) to 30%.

Hydrogen peroxide (H_2_O_2_) is a powerful oxidizing agent that facilitates the detachment and loosening of the lignocellulosic material, thus increasing its accessibility. H_2_O_2_ is also capable of penetrating the amorphous domains of the cellulosic materials and promotes degradation of lignin and decolorization [43]. This study investigated the effect of H_2_O_2_ concentration, ranging from 5 to 30% on the pre-treatment of BP powder. BP powder suspended in deionized water (0% H_2_O_2_) was used as a control. As shown in Figure 3, pre-treatment with increasing H_2_O_2_ concentration resulted in BP decolorization from dark brown (control) to light yellow. According to Andrade et al. [44], the dark brown color of lignocellulosic material is due to the presence of chromogen groups such as conjugated carbonyls, double bonds, and their combination. The color change of the suspension following pre-treatment indicates the partial degradations of hemicellulose and lignin substances [31].

Several reactions may occur during the oxidation of lignocellulosic biomass by hydrogen peroxide, including the cleavage of alkyl aryl ether linkages, displacement of side chains, electrophilic substitution, and oxidative cleavage of aromatic nuclei. Dissociation of H_2_O_2_ generated hydroperoxide anion (HOO^−^), which reacted with undissociated H_2_O_2_ to form highly reactive hydroxyl (HO•) and superoxide (O_2_−•) radicals. HOO^−^ is responsible for lignin oxidation, preferentially attacking the ethylenic and carbonyl groups, whereas HO• and O_2_−• attack the lignin sidechains to produce water-soluble oxidation that promotes decolorization. In this process, most of the lignin and hemicellulose components were degraded simultaneously. A combination of H_2_O_2_ pre-treatment with other treatments to eliminate lignin and hemicellulose were studied by some researchers, such as enzymatic [45], alkali [46], and microwave-assisted dilute alkali [42] pre-treatments. These works have demonstrated that H_2_O_2_ pre-treatment plays a significant role in the hydrolysis of lignocellulosic biomass.

#### 3.2.2. Effect of Sulfuric Acid Concentration on the Hydrolysis of BP Powder

Sulfuric acid hydrolysis is the most common method used for producing nanocellulose, owing to the high uniformity, crystallinity, and stability of the nanocellulose produced. However, high acid concentration, typically 64 wt% H_2_SO_4_, has poor selectivity over amorphous and crystalline regions, resulting in the degradation of some of the crystalline phases. This decreases the hydrolysis performance, especially when it is isolated from lignocellulosic biomass with a low cellulose content. Therefore, dilute acid treatment appears to be the preferred method for milder hydrolysis reaction. The efficacy of sulfuric acid hydrolysis can be observed based on the color change of the suspension from brown to white [47]. In this study, dilute sulfuric acid with a concentration ranging from 0 to 10% was used to hydrolyze the pre-treated banana peel. The control sample (0%) appears slightly yellowish, indicating the presence of a small amount of lignin and hemicellulose that decreased gradually during acid hydrolysis (Figure S1). White suspension was obtained at 6% concentration, signifying that most of the impurities have been removed from the sample. A further increase in the acid concentration to 8% and 10% may cause overdegradation of the banana peel, as evident by the slight brownish hue of the suspensions [47].

Cellulose microfibril in BP fiber contains crystalline and amorphous domains that are randomly distributed along their length [48]. The cellulose chain in the crystalline domain is packed closely, while in the amorphous domain, it is arranged in a disordered manner and is more susceptible to hydrolytic action. During acid hydrolysis, the H^+^ ions were able to penetrate the amorphous regions and facilitate hydrolytic cleavage of *β*-1,4 glycosidic bonds in cellulose chain, and produced CNCs [16]. According to Lee et al. [49], the formation of CNCs through H_2_SO_4_ hydrolysis involved the following mechanism: (i) Protonation of the oxygen atom of glycosidic bond; (ii) Replacement of unstable positive charged group in the cellulose chain by hydroxyl group of water; and (iii) Esterification between H_2_SO_4_ and hydroxyl group, producing negatively charged CNCs with sulfate ester groups (–OSO_3_^−^). 

### 3.3. FTIR Analysis

**Figure 4:**
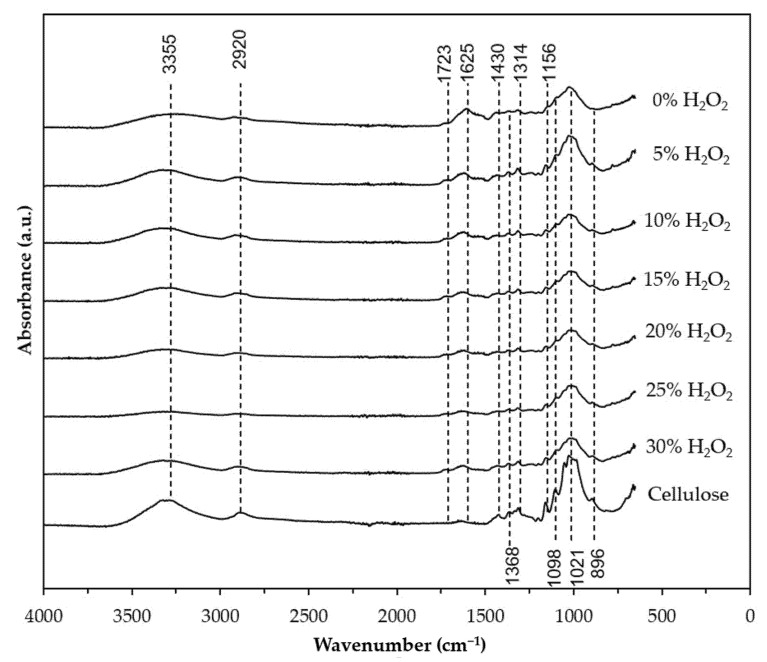
FTIR spectra of the banana peel after pre-treatment using different H_2_O_2_ concentration (5%, 10%, 15%, 20%, 25%, and 30%). Untreated banana peel (0% H_2_O_2_) was used as a control. The pre-treatment was conducted at 90 °C for 2 h.

**Figure 5:**
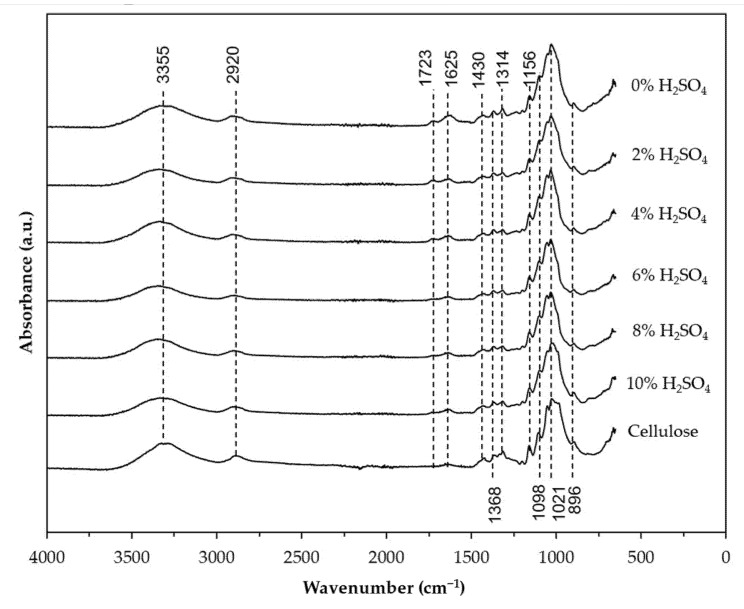
FTIR spectra of nanocellulose obtained after hydrolysis with of the H_2_O_2_ pre-treated banana peel using different H_2_SO_4_ concentration (2%, 4%, 6%, 8%, and 10%). H_2_O_2_ pre-treated banana peel without the addition of H_2_SO_4_ (0% H_2_SO_4_) was used as a control The hydrolysis reaction was conducted at 80 °C for 1 h.

Figure 4 and Figure 5 depict the FTIR spectra of the BP in response to pre-treatment and hydrolysis, with varying concentrations of H_2_O_2_ and H_2_SO_4_. All samples exhibit two major absorbance regions at the wavenumbers 3700–2900 cm^−1^ and 1700–800 cm^−1^. The broad absorption band in the 3700–3000 cm^−1^ region corresponds to the exposed OH groups of cellulose, which are bound by intermolecular hydrogen bonding [50,51]. The similarity of the absorption band between the banana peel sample and the H_2_O_2_ and H_2_SO_4_-treated samples at this region is a clear indication that the crystalline cellulose in the raw materials was not disrupted during the nanocellulose isolation process [52]. The band at 2920 cm^−1^ is assigned to asymmetrical stretching vibration of the C–H groups in polysaccharides [16].

As shown in Figure 4, the banana peel spectrum (0% H_2_O_2_) displayed a peak at 1723 cm^−1^ attributed to the stretching vibration C=O ester bonds of ferulic and/or p-coumaric acids, which are bonded together with the hemicellulose [31]. This peak is not present in the reference spectra and became less intense in the H_2_O_2_ pre-treated samples. Huang et al. [42] reported a similar observation when rape raw residue was subjected to H_2_O_2_ treatment. Similarly, the peak at 1625 cm^−1^, which originated from stretching of the C=C and C=O lignin aromatic ring [53], gradually reduced with increasing H_2_O_2_ concentration. Gradual disappearance of these peaks suggests that the hemicellulose and lignin have been partially removed from the banana peel. Based on this finding and the color change observed in Figure 3, 30% H_2_O_2_ was selected for further investigation on acid hydrolysis. An obvious reduction in the peak intensity at 1723 and 1625 cm^−1^ can be observed when the pre-treated sample was treated further with H_2_SO_4_ (Figure 5). Indeed, the absence of the peak at 1723 cm^−1^ in samples treated with 6%, 8%, and 10% of H_2_SO_4_ suggests that the hemicellulose and lignin components that are present in the banana fiber were almost entirely eliminated during the acid hydrolysis at these conditions [54]. The success of the one-pot H_2_O_2_ pre-treatment and H_2_SO_4_ hydrolysis was further confirmed by the intensified absorption bands at 1430, 1368, 1314, and 1021 cm^−1^ and the appearance of peak at 896 cm^−1^ at all the H_2_O_2_/H_2_SO_4_-treated samples, which corresponds to stretching and bending vibrations of –CH_2_ and –CH, –OH and C–O bonds in cellulose [55,56]. These peaks did not appear or were less intense in the banana peel spectrum.

### 3.4. Crystallinity of the CNCs

**Figure 6:**
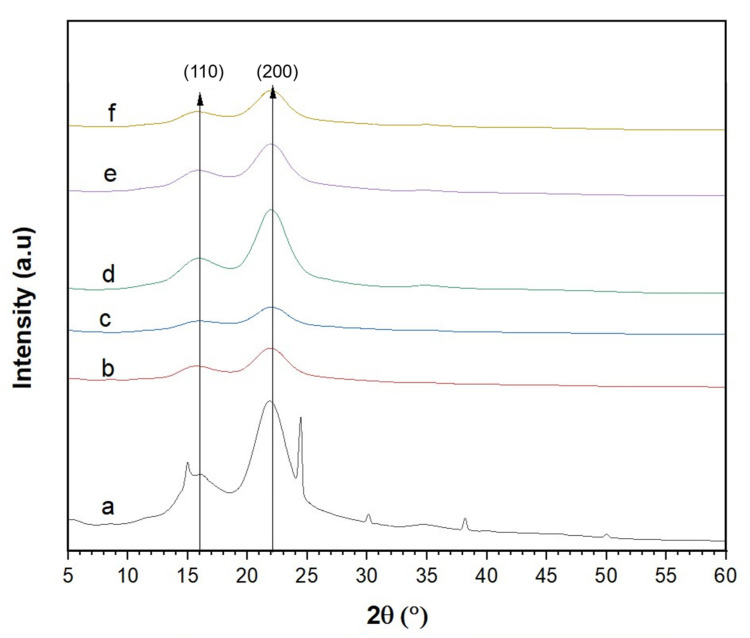
X-ray diffraction pattern of the nanocellulose obtained after hydrolysis with different sulfuric acid concentrations: (**a**) 0%, (**b**) 2%, (**c**) 4%, (**d**) 6%, (**e**) 8%, and (**f**) 10%.

Crystallinity of CNCs is one of the main factors that determines its mechanical strength, thermal degradation behavior, and reinforcing ability in nanocomposite applications [57]. Figure 6 shows that all the hydrolyzed samples have a similar XRD pattern, with prominent peaks at 2θ = 16° and 22° corresponding to the (110) and (200) lattice planes which represent the typical cellulose I [10,47,58]. The findings are consistent with other research [9,52,55], confirming that the original structure of native banana peel cellulose was retained after the isolation process [55]. Additional peaks that appeared in the spectra of the control sample (0% H_2_SO_4_) indicate the presence of impurities, probably from an inorganic substance as reported by [59].

From Table 3, it was observed that the H_2_SO_4_ concentration influenced the degree of crystallinity. The crystallinity of BP powder was determined to be 58.1%, and the value increase after H_2_O_2_ pre-treatment and H_2_SO_4_ hydrolysis due to the elimination of hemicellulose and delignification process [9], as well as the removal of amorphous domains of cellulose [60]. The sample hydrolyzed with 6% H_2_SO_4_ had the highest crystallinity of 85.2%, comparable to that of the standard cellulose (88.7%) and CNCs isolated from enset fiber (80.9%) [51], pear fruit peel (85.7%) [7], and date palm trunk mesh (89.6%) [61], but much higher than those from pea hull waste (77%) [58], Spanish poplar biomass (65%) [62], and cocoa pod husk (67.6%) [54]. The high crystallinity and sharp diffraction peak indicate a stronger hydrogen bonding interaction between the nanocellulose chains, which creates a high crystalline and compact structure [63]. However, lower crystallinity was obtained when the banana peel samples were hydrolyzed with 8% and 10% H_2_SO_4_. This could be due to partial degradation of crystalline region of the cellulose fiber [52] and is in agreement with the color changes and FTIR data.

### 3.5. SEM, TEM, and AFM Analyses

**Figure 7:**
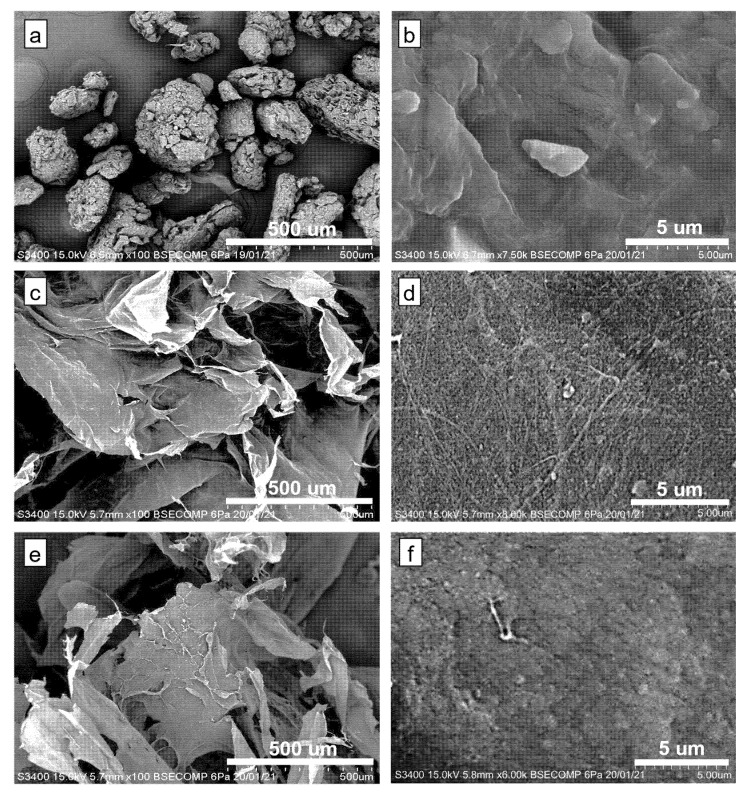
SEM images of (**a**,**b**) Banana peel powder; (**c**,**d**) Freeze-dried H_2_O_2_-pre-treated banana peel; and (**e**,**f**) Freeze-dried H_2_O_2_/ H_2_SO_4_-treated banana peel. The concentrations of H_2_O_2_ and H_2_SO_4_ used were 30% and 6%, respectively.

The microstructure of the BP powder and the freeze-dried suspensions after the one-pot process were observed using Scanning Electron Microscope (SEM). As presented in Figure 7, the raw BP appears as aggregated structure with a rough surface due to the intact lignocellulosic components. Clear morphological changes were observed after the BP powder was subjected to pre-treatment and hydrolysis. The freeze-dried suspensions showed a sheet-like structure, which was commonly observed when cellulose suspension was subjected to a relatively slow freezing process (−20 °C) [64]. Closer observation on the surface of the sheet revealed randomly arranged nanosized cellulose fibers with a diameter ranging from 70–150 nm. Further hydrolysis with 6% H_2_SO_4_ causes fragmentation of the cellulose fibers, as can be seen in Figure 7f.

The formation of a sheet-like structure during freeze-drying of cellulose suspension could be associated with the self-assembly behavior of cellulose, brought about by the strong hydrogen bonding between cellulose sub-units. Extensive investigation on the self-assembly behavior of cellulose nanoparticles during freeze-drying has been conducted by [65]. In their study, CNCs and CNFs were extracted from bleached wood pulp by pre-treatment with 20 wt% aqueous sodium hydroxide solution followed by hydrolysis with 64% and 48% sulfuric acid, respectively. It was inferred that the occurrence of this phenomenon could be related to the basic physics of ice crystal growth, as well as the interaction between cellulose particles. The freezing process causes the cellulose particles to concentrate and become trapped at the edge of the growing ice crystals. The particles were then aggregated with the adjacent particles through hydrogen bonding and were further rearranged and self-assembled along the freezing direction to form a sheet-like structure. Similar observations were also reported when walnut shells [66] and empty fruit bunches [64] were subjected to freeze-drying.

**Figure 8:**
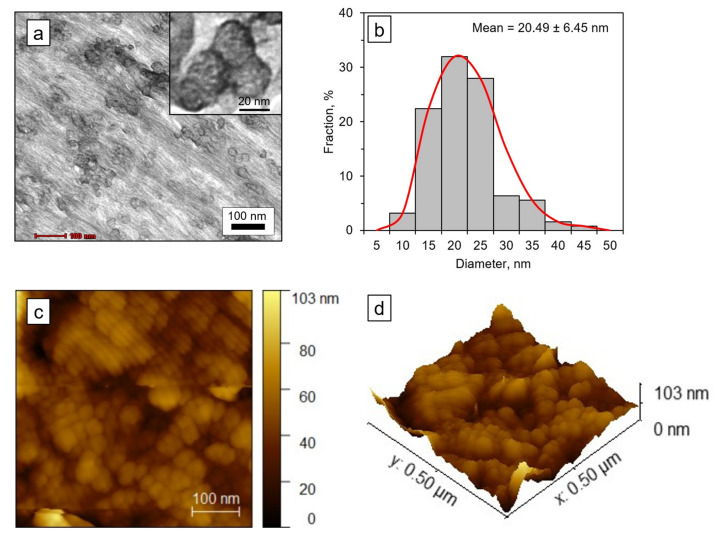
Morphological properties of CNCs obtained after one-pot microwave and oxidative hydrolysis treatment with 30% H_2_O_2_ and 6% H_2_SO_4_: (**a**) TEM image; (**b**) Particle size distribution of nanocellulose analyzed from the TEM images using ImageJ; (**c**,**d**) AFM images.

The TEM and AFM images in Figure 8 confirmed the presence of spherical CNCs particles with uniform size distribution and an average size of 20.5 ± 6.5 nm. The particle morphology is different from the typical rod- or needle-like morphologies of CNCs obtained through sulfuric acid hydrolysis [54,60,67,68]. Although several studies have reported the production of spherical CNCs with a size of less than 100 nm from various sources, including waste cotton [52,69], oil palm empty fruit bunch [70], and pear fruit peel [7], the exact structure and formation mechanism are principally unknown. Chen et al. [7] postulated that the formation of spherical CNCs could be attributed to the self-assembly of short cellulose rods via interfacial hydrogen bonds. Ultrasonic treatment applied to the suspension prior to the TEM and AFM analyses might also facilitate the formation of spherical CNCs, as reported by Azrina et al. [70]. According to Dong et al. [71], spherical CNCs could be potential candidates as a Pickering stabilizer in food, cosmetic, and biomedicine due to their high emulsification index, high stability, and low viscosity.

Aggregation of the CNCs can also be clearly observed in both the TEM and AFM images which led to the formation of a dense layered structure (Figure 8d). During the drying process of the suspension, the CNCs particles become closer to one another as the water is removed, and finally form a close-packed network [72]. This phenomenon is possibly driven by a layer-by-layer and thermodynamically favored process to reduced specific surface area [7]. Besides that, the smaller distance between the particles could also be due to the effect of capillary and diffusion forces [70].

### 3.6. Particle Size and Zeta Potential Analyses

**Figure 9:**
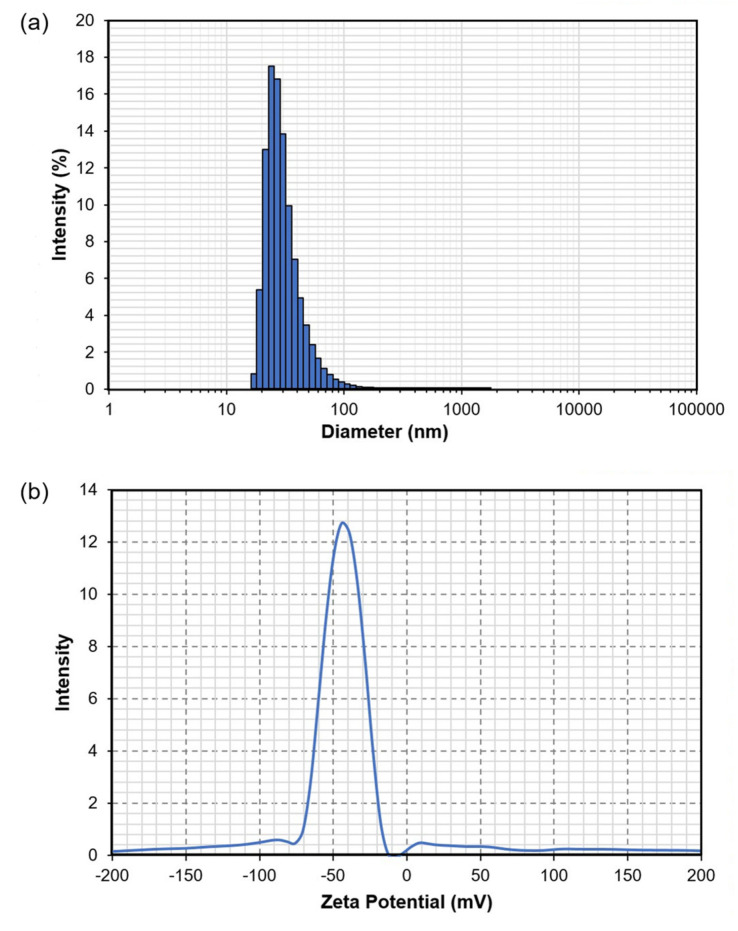
Distribution of (**a**) particle size and (**b**) Zeta potential of CNCs produced from banana peel using the one-pot process.

Dynamic light scattering (DLS) is one of the most frequently used methods to obtain the size of nanoparticles dispersed in liquids. The particle size distribution of the banana peel CNCs in aqueous suspension is shown in Figure 9a. Narrow particle size distribution can be observed with an average diameter of 89.9 ± 23.9 nm. About 77% of the particles had a size below 100 nm, and 99.5% were less than 200 nm. It should be noted that the particle size obtained by DLS is about four times larger than the size measured from TEM image, a tendency that have been reported previously by Gong et al. [73] and Harini et al. [8]. This could be due to the fact that DLS measures the hydrodynamic diameter of the particles, which also includes the hydration layer, leading to a larger particle size [74]. Besides that, the presence of bigger particles may contribute to an increase in light scattering, shifting the measured particles’ size towards larger values [75].

Zeta potential is an important parameter that relates to the surface charge of particles and determines the stability of CNCs suspension [31]. High zeta potential (absolute value) indicates stable suspension and low tendency of particle aggregation. On the other hand, for suspension with low zeta potential, repulsive forces between the particles are minimal, which may lead to the formation of aggregates [68]. Figure 9b shows the zeta potential distribution of the CNCs suspension obtained after the one-pot process. The CNCs exhibit a high negative surface charge (−42.9 mV), consistent with previous reports on CNCs produced through H_2_SO_4_ hydrolysis [52,68,76]. Generally, particles with absolute zeta potential of higher than 30 mV are considered kinetically stable [52]. The high zeta potential of the CNCs is due to the formation of anionic sulfate ester groups (–OSO_3_−) during acid hydrolysis with H_2_SO_4_, which results in strong electrostatic repulsion [76].

## 4. Conclusions

The present study provides insight on the feasibility of one-pot oxidative hydrolysis system using hydrogen peroxide and sulfuric acid at mild concentrations for isolation of nanocellulose from abundant and underutilized banana peels. Spherical CNCs with average size of 20.5 ± 6.5 nm, high crystallinity, and good colloidal stability was successfully isolated from banana peel powder. The method employed could provide simpler and environmentally benign alternatives to the conventional nanocellulose isolation method. Investigation of swelling, mechanical, and thermal properties of the CNCs should be explored in future studies for potential food applications such as additives and stabilizers, as well as reinforcement in food packaging materials.

## Figures and Tables

**Table 1:** Proximate compositions of BP powder.

Banana Variety	% Dry Weight				Reference
	Moisture	Ash	Fat	Protein	
Saba (*Musa acuminata* × *balbisiana*)	8.2 ± 0.2	13.8 ± 0.3	12.0 ± 0.2	5.4 ± 0.1	Present study *
*Musa acuminata*	10.0 ± 0.9	15.6 ± 0.3	10.4 ± 0.4	10.6 ± 0.2	[37]
*Musa sapientum*	15.5–17.8	11.3–14.7	2.4–3.3	2.2–2.7	[35]
*Musa sapientum* Linn. cv. Mali-On	7.7	12.6	4.3	3.7	[29]
*Musa sapientum*	50.5 ± 2.7	8.8 ± 0.5	1.6 ± 0.1	5.3 ± 0.02	[36]

* Data are mean values of three replicates ± standard deviation.

**Table 2:** Chemical compositions of BP powder.

Banana Variety	% Dry Weight			Reference
	Cellulose	Hemicellulose	Lignin	
Saba (*Musa acuminata* × *balbisiana*)	12.1 ± 0.3	14.8 ± 0.9	15.7 ± 2.1	Present study *
n.a.	11.5	25.5	9.8	[19]
Terra (*Musa paradisiaca*)	12.1	10.2	2.9	[15]
Terra (*Musa paradisiaca*)	7.5	-	7.9	[14]
*Mpologoma*, *Kisansa* and *Kibuzi*	9.9 ± 0.1	41.4 ± 0.8	8.9 ± 1.3	[17]
Cavendish	18.7 ± 3.2	20.3 ± 7.4	16.8 ± 4.6	[18]
*Musa ABB*	15.4 ± 1.5	4.0 ± 1.0	3.2 ± 0.8	[20]

n.a. not available; * Data are mean values of three replicates ± standard deviation.

**Table 3:** Crystallinity index (CrI) of raw banana peel and after hydrolysis with 0 to 10% sulfuric acid.

Sulfuric Acid Concentration (%)	Crystallinity Index (CrI)
BP powder	58.1
0	64.0
2	65.6
4	64.1
6	85.2
8	77.8
10	71.7
Avicel^®^ PH-101	88.7

## Data Availability

Not applicable.

## Supplementary Materials

The following supporting information can be downloaded at: https://www.mdpi.com/article/10.3390/nano12193537/s1, Figure S1: Suspensions obtained after the H_2_O_2_-pre-treated banana peel samples were subjected to acid hydrolysis at concentration ranging from 0% (control) to 10%.
