# Supplementary material for: Isolation of Cellulose Nanocrystals from Banana Peel Using One-Pot Microwave and Mild Oxidative Hydrolysis System
Source: Nanomaterials (Basel). 2022 Oct 10;12(19):3537. doi: 10.3390/nano12193537 (PMC9565709; doi:10.3390/nano12193537)
Supplement: Supplementary file 1 [file nanomaterials-12-03537-s001.zip › nanomaterials-1904047-supplementary.pdf]

Supplementary Materials

Isolation of Cellulose Nanocrystals from Banana Peel Using One-Pot Microwave and Mild Oxidative Hydrolysis System

Nurhidayah Azmirah Mohd Jamil [1,2], Syafiqah Syazwani Jaffar [1], Suryani Saallah [1,*], Mailin Misson [1], Shafiquzzaman Siddiquee [1], Jumardi Roslan [3] and Wuled Lenggoro [4]

1 Biotechnology Research Institute, Universiti Malaysia Sabah, Jalan UMS, Kota Kinabalu 88400, Sabah, Malaysia
2 Marine Aquaculture Development Centre Menggatal, Department of Fisheries Sabah, Jalan Sepanggar, Kota Kinabalu 88450, Sabah, Malaysia
3 Faculty of Food Science and Nutrition, Universiti Malaysia Sabah, Jalan UMS, Kota Kinabalu 88400, Sabah, Malaysia
4 Institute of Engineering, Tokyo University of Agriculture and Technology, 2-24-16 Nakacho, Koganei, Tokyo 184-8588, Japan
* Correspondence: suryani@ums.edu.my

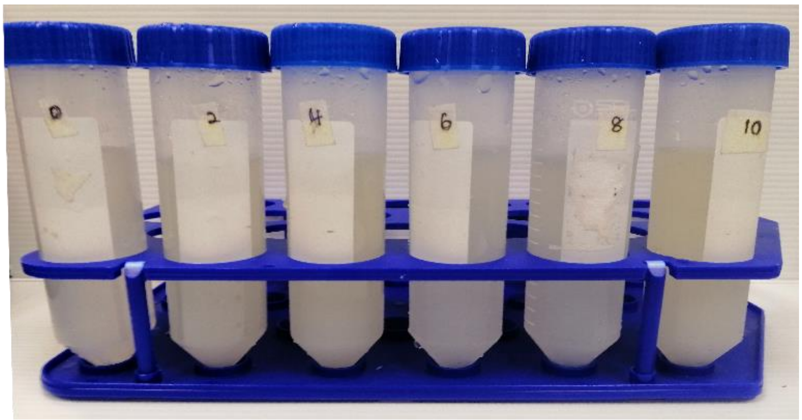

Figure S1. Suspensions obtained after the H_2O_2-pre-treated banana peel samples were subjected to acid hydrolysis at concentration ranging from 0% (control) to 10%.
